# Membrane potential hyperpolarization: a critical factor in acrosomal exocytosis and fertilization in sperm within the female reproductive tract

**DOI:** 10.3389/fcell.2024.1386980

**Published:** 2024-05-13

**Authors:** Paula A. Balestrini, Valeria Sulzyk, Martina Jabloñski, Liza J. Schiavi-Ehrenhaus, Soledad N. González, Juan J. Ferreira, Matías D. Gómez-Elías, Pablo Pomata, Guillermina M. Luque, Dario Krapf, Patricia S. Cuasnicu, Celia M. Santi, Mariano G. Buffone

**Affiliations:** ^1^ Instituto de Biología y Medicina Experimental (IByME)-Consejo Nacional de Investigaciones Científicas y Tícnicas, Buenos Aires, Argentina; ^2^ Department of OB/GYN, Washington University School of Medicine, Saint Louis, MO, United States; ^3^ Instituto de Biología Molecular y Celular de Rosario, Consejo Nacional de Investigaciones Científicas y Técnicas–Universidad Nacional de Rosario, Rosario, Santa Fe, Argentina

**Keywords:** fertilization, capacitation, sperm, oviduct, mating, hyperpolarization, acrosomal exocytosis

## Abstract

Hyperpolarization of the membrane potential (Em), a phenomenon regulated by SLO3 channels, stands as a central feature in sperm capacitation—a crucial process conferring upon sperm the ability to fertilize the oocyte. *In vitro* studies demonstrated that Em hyperpolarization plays a pivotal role in facilitating the mechanisms necessary for the development of hyperactivated motility (HA) and acrosomal exocytosis (AE) occurrence. Nevertheless, the physiological significance of sperm Em within the female reproductive tract remains unexplored. As an approach to this question, we studied sperm migration and AE incidence within the oviduct in the absence of Em hyperpolarization using a novel mouse model established by crossbreeding of SLO3 knock-out (KO) mice with EGFP/DsRed2 mice. Sperm from this model displays impaired HA and AE *in vitro*. Interestingly, examination of the female reproductive tract shows that SLO3 KO sperm can reach the *ampulla*, mirroring the quantity of sperm observed in wild-type (WT) counterparts, supporting that the HA needed to reach the fertilization site is not affected. However, a noteworthy distinction emerges—unlike WT sperm, the majority of SLO3 KO sperm arrive at the *ampulla* with their acrosomes still intact. Of the few SLO3 KO sperm that do manage to reach the oocytes within this location, fertilization does not occur, as indicated by the absence of sperm pronuclei in the MII-oocytes recovered post-mating. *In vitro*, SLO3 KO sperm fail to penetrate the ZP and fuse with the oocytes. Collectively, these results underscore the vital role of Em hyperpolarization in AE and fertilization within their physiological context, while also revealing that Em is not a prerequisite for the development of the HA motility, essential for sperm migration through the female tract to the *ampulla*.

## Introduction

During their transit through the oviduct, sperm undergo a series of physiological and biochemical changes that are largely englobed under the term capacitation (Austin, 1951; Chang, 1951). This allows sperm to acquire two fundamental cellular events necessary for fertilization. First, to develop a special pattern of motility called hyperactivation (HA), which is essential for sperm migration in the female reproductive tract (Suarez and Pacey, 2006; Ho et al., 2009). Second, sperm acquire the capacity to undergo a regulated acrosomal exocytosis (AE), a unique type of secretion where the acrosome, a single vesicle, is secreted after stimulation without any membrane recycling, rendering this event irreversible (Mayorga et al., 2007). AE is required for re-localization of proteins responsible for sperm-oocyte fusion, making this event indispensable for fertilization (Inoue et al., 2005; Satouh et al., 2012).

At the molecular level, hyperpolarization of the membrane potential (Em) takes place during capacitation, and is fundamental for both, HA and AE (De La Vega-Beltran et al., 2012; Torrezan-Nitao et al., 2020; Balestrini et al., 2021). Several mechanisms have been proposed to regulate sperm Em including the CFTR, ENaC and SLO1 channels (Santi et al., 2010; Mannowetz et al., 2013; Puga Molina L. D. et al., 2018; Puga Molina L. C. et al., 2018). So far in both mice and humans, the sperm specific potassium (K^+^) ion channel, SLO3, seems to be the principal regulator of sperm Em (Santi et al., 2010; Zeng et al., 2011; Brenker et al., 2014; Lyon et al., 2023). SLO3 knock-out (KO) mice are sterile; sperm from these mice do not hyperpolarize their Em during capacitation, have impaired sperm motility, do not undergo AE, and cannot fertilize *in vitro* (Santi et al., 2010; Zeng et al., 2011). Pharmacological hyperpolarization using valinomycin, a K^+^ ionophore, enables cells that would not undergo AE (such as non-capacitated and SLO3 KO mice sperm cells) to respond to stimuli and therefore induces exocytosis (Santi et al., 2010; De La Vega-Beltran et al., 2012; Balestrini et al., 2021). Both loss-of function (SLO3 KO mouse) and gain-of function (valinomycin) experiments demonstrated that Em hyperpolarization is necessary and sufficient to prepare cells to induce AE. In humans, the importance of Em hyperpolarization was recently associated with the success rate in *in vitro* fertilization (IVF) (Baro Graf et al., 2020; Puga Molina et al., 2020). It has also been shown that specific inhibitors of human SLO3 channels impaired human sperm HA motility, AE and Em hyperpolarization (Lyon et al., 2023).

Both HA motility and AE require calcium (Ca^2+^) entry (Ren et al., 2001; Kirichok et al., 2006; Mayorga et al., 2007; Lishko and Kirichok, 2010; Lucchesi et al., 2016). However, how sperm membrane hyperpolarization regulates this Ca^2+^ influx is not completely understood. HA motility requires the activation of the sperm specific and pH-activated Ca^2+^ channel CatSper (Ren et al., 2001; Kirichok et al., 2006; Ho et al., 2009; Lishko and Kirichok, 2010). In this regard, SLO3 KO sperm showed impaired Ca^2+^ entry though CatSper (Chávez et al., 2013; Balbach et al., 2020) indicating a functional relationship between these two channels. It has been suggested that SLO3 hyperpolarization contributes to the activation of CatSper channels by increasing intracellular pH (pH_i_) through the activation of a sperm specific and putative hyperpolarization-activated Na^+^/H^+^ exchanger (sNHE) (Wang et al., 2007; Chávez et al., 2014; Windler et al., 2018; de Prelle et al., 2022). Alternatively, the hyperpolarization induced by SLO3 could increase the driving force of Ca^2+^, contributing to Ca^2+^ influx through the weak voltage-dependent CatSper channel.

Despite the numerous papers that clearly demonstrate the critical role of Em hyperpolarization in sperm function, little is known about this phenomenon in the physiological context: the female reproductive tract. Most of the available data was collected from *in vitro* experimentation, where sperm cells are obtained from the epididymis and capacitation occurs *in vitro*. These experimental settings may not necessary reflect what takes place *in vivo*. Thus, in this study we generated a new mouse model by crossing SLO3 KO female mice with mice that expresses DsRed2 in the midpiece, and EGFP in the acrosome (Hasuwa et al., 2010; Santi et al., 2010). This new model allowed us to study the role of Em hyperpolarization *in vivo*. Specifically, we investigated two previously unresolved matters: 1) the impact of Em on sperm migration within the oviduct, and 2) the significance of Em hyperpolarization for sperm acrosomal exocytosis (AE) *in vivo*. In this context, we assessed the relevance of AE in the upper segments of the oviduct, given that sperm lacking SLO3 do not undergo AE.

## Results

### Transgenic EGFP/DsRed2 sperm lacking SLO3 channels fail to undergo acrosomal exocytosis

To study the importance of Em hyperpolarization *in vivo*, we generated a new transgenic mouse model by mating SLO3 KO female mice with EGFP/DsRed2 transgenic male mice. These mice allowed us to follow sperm migration within the female reproductive tract *in vivo*, by visualizing midpiece mitochondria using DsRed2 fluorescence and the acrosomal status using EGFP as a marker.

**FIGURE 1:**
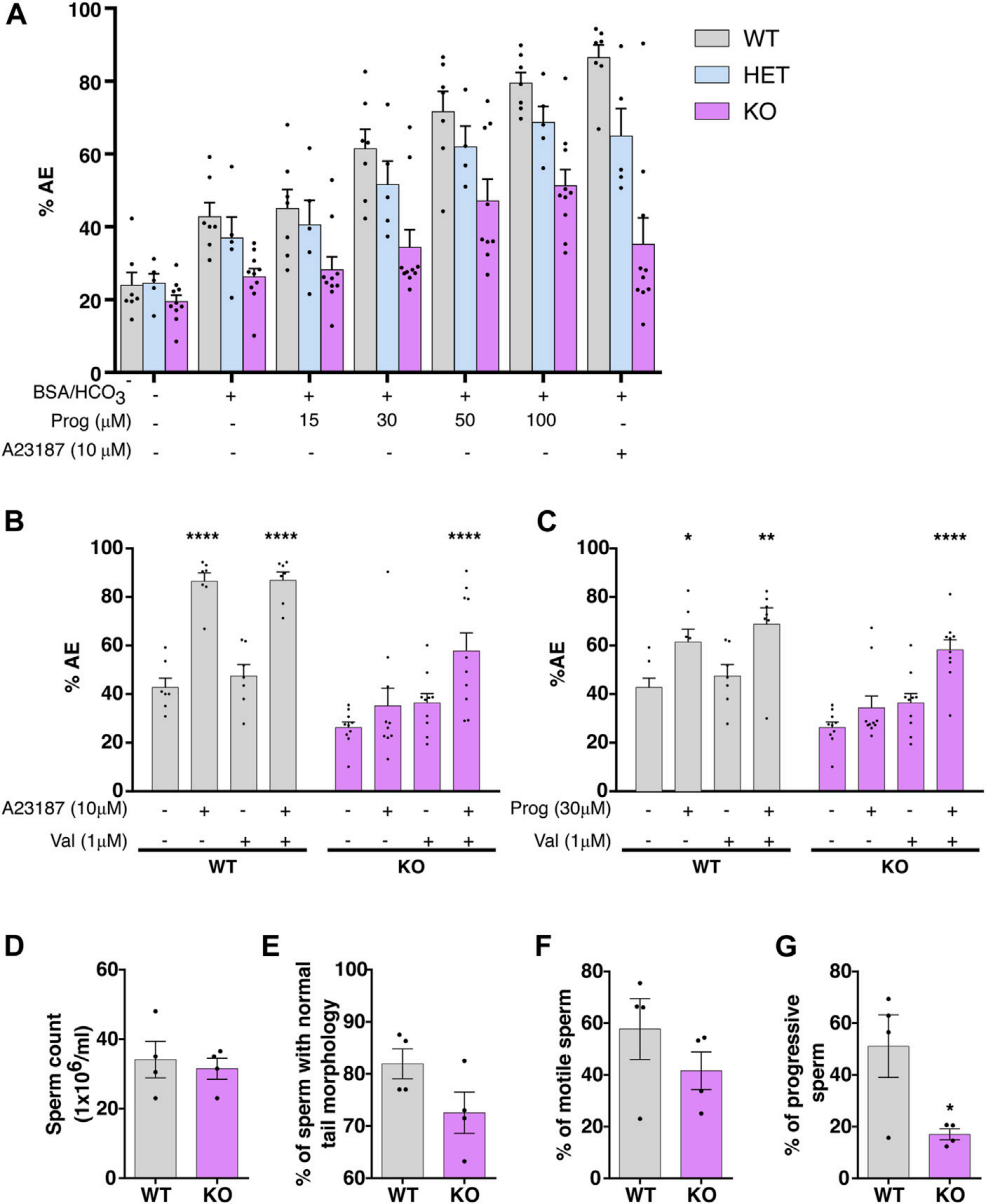
Validation of the EGFP-SLO3 mouse model. **(A)** Live sperm from wild-type (WT), heterozygote (HET) or knock-out (KO) mice were incubated for 60 min in the presence or absence of BSA/HCO_3_^−^ medium and challenged with increasing concentrations of progesterone (Prog) to induce AE for 30 min. As a control, 10 μM of A23187 was used. The percentage of live AE sperm was analyzed by flow cytometry. Values represent the mean ± SEM of at least four different experiments (N ≥ 4; 7 WT, 4 HET and 9 KO males). **(B,C)** Sperm from WT or KO mice were incubated for 60 min in capacitating (CAP: BSA/HCO_3_^−^) medium in the presence or absence of 1 μM valinomycin (Val) and AE was induced using 10 μM A23187 **(B)** or 30 μM Prog **(C)**. Values represent the mean ± SEM of at least seven different experiments (N ≥ 7, 7 WT and 10 KO males). ****p < 0.0001, **p < 0.01, *p < 0.05 represent statistical difference. Two-way ANOVA in combination with Dunnet’s multiple comparisons test was performed against the control condition (CAP). **(D–G)** Sperm from WT or KO mice were allowed to swim-Out and the percentage of normal sperm tail morphology was observed in live sperm **(E).** Values represent the mean ± SEM of four different experiments (*N* = 4; 4 WT and 4 KO males). Paired t-test, and Non-parametric Wilcoxon-Mann-Whitney test were performed for sperm count and morphology respectively. **(F,G)** The percentage of motile and progressive sperm was analyzed using CASA. Data represents the mean ± SEM of four different experiments (*N* = 4; 4 WT and 4 KO males). Non-parametric Wilcoxon-Mann-Whitney test was performed.

To validate the new transgenic model, the occurrence of AE was evaluated in EGFP/DsRed2-SLO3 WT, HET and KO mice sperm *in vitro* by flow cytometry. As shown in Figure 1A, AE was induced in capacitated sperm using increasing concentrations of progesterone (Prog) or a single concentration of Ca^2+^ ionophore A23187 (10 µM). Results show that WT sperm displayed a concentration-dependent increase in AE in response to progesterone. In contrast, KO sperm did not display an increase in AE at low progesterone concentrations (15–30 µM). Although at higher concentrations (50–100 µM), KO sperm showed an increase in the AE rate, in both concentrations the percentage of AE was always lower when compared to WT sperm. HET mice presented an intermediate phenotype (Figure 1A) thus, for all the following experiments, the WT mice were used as control. As previously reported, exposure of WT sperm to A23187 significantly increased the percentage of acrosome reacted cells while it did not in sperm from KO mice (Santi et al., 2010).

To further assess this new mouse model, sperm were capacitated in the presence or absence of Valinomycin (Val) to induce pharmacological hyperpolarization of the Em. When treated with 10 µM A23187 (Figure 1B) or 30 µM Prog (Figure 1C), sperm from WT male mice undergo AE in the presence or absence of Val. In contrast, sperm from KO mice only increase the level of AE when pharmacological hyperpolarization was previously induced. Altogether, these results support previous findings where Em hyperpolarization is necessary for sperm to undergo AE. Similar results were observed previously by inducing AE with A23187 (Santi et al., 2010). Here, we evaluated AE only in the live sperm population, by flow cytometry and studied this process using Prog, a more physiological inductor than A23187.

No significant differences were found in sperm count or in the percentage of morphologically normal sperm between WT and KO (Figures 1D, E), despite that KO sperm displayed a tendency towards a higher incidence of hairpin flagellar morphology after swim-out. As previously described (Santi et al., 2010), sperm obtained after swim-out from KO male mice had significantly impaired progressive motility when compared to sperm from WT mice (Figures 1F, G.

### Sperm lacking SLO3 channels are unable to develop HA *in vitro* despite undergoing pH alkalinization

**FIGURE 2:**
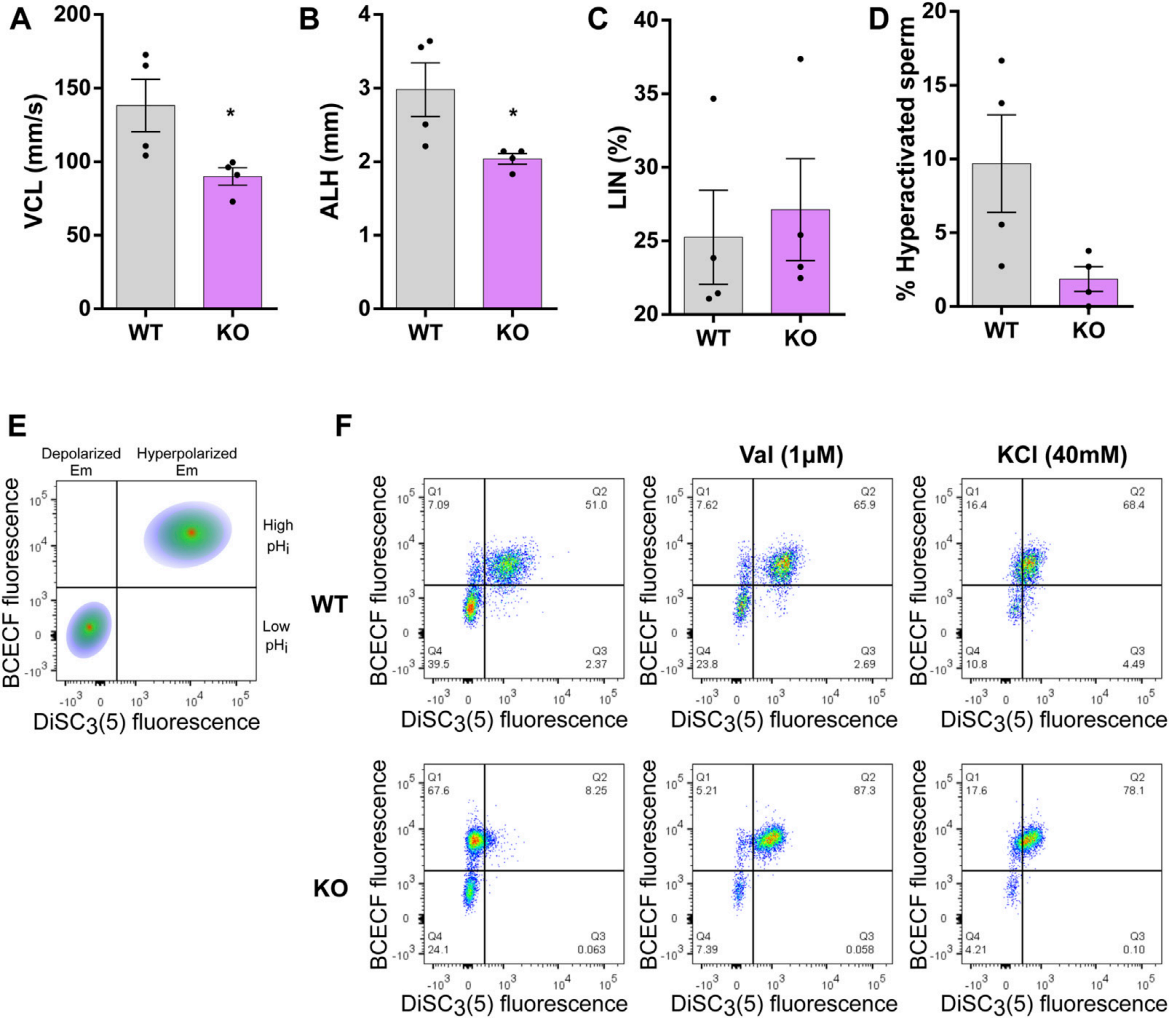
Assessment of HA, pH_i_, and Em in WT and KO sperm. Live sperm from WT or KO mice where capacitated for 60 min. **(A–D)** The curvilinear velocity (VCL), amplitude of lateral head displacement (ALH), linearity (LIN) and Hyperactivation (HA) were assessed using CASA. Non-parametric Wilcoxon-Mann-Whitney test was performed to evaluate differences between WT and KO. *p < 0.05 stands for statistical difference between WT and KO. Data represents the mean ± SEM of four different experiments (*N* = 4; 4 WT and 4 KO males). **(E,F)** To evaluate pH_i_, cells were washed and incubated for 20 min with 5 µM BCECF-AM, then washed again and finally incubated with 1 nM DiSC_3_(5) and 1 µg/mL PI in order to evaluate sperm Em and viability using flow cytometry. **(E)** A schematic dot-plot of BCECF fluorescence vs. DiSC_3_(5) fluorescence is shown. **(F)** Representative dot-plots of BCECF fluorescence vs. DiSC_3_(5) fluorescence for WT and KO sperm. Capacitated WT and KO sperm were incubated with 1 µM valinomycin for 5 min, data was collected and addition of 40 mM KCl was used. 5 different experiments were performed (*N* = 5; 5 WT and 5 KO males).

Sperm require HA motility to migrate in the oviduct, specially to escape from the oviductal sperm reservoir and reach the oocyte (Demott and Suarez, 1992; Ho et al., 2009). Thus, we also evaluated the ability of SLO3 KO sperm to develop HA motility *in vitro*. Sperm from WT and KO mice were incubated under capacitating conditions and the HA was evaluated using computer assisted sperm analysis. As shown in Figure 2, SLO3 KO sperm presented a significant decrease in kinetic parameters directly associated with HA motility: curvilinear velocity (VCL) and lateral head displacement (ALH). No differences were observed in linearity (LIN) (Figures 2A–C). As a result, the percentage of sperm from KO mice exhibiting HA motility was reduced compared to WT sperm (Figure 2D).

It has been proposed that the absence of Em hyperpolarization in SLO3 KO impedes the development of HA by decreasing Ca^2+^ influx through CatSper channels. There is evidence that SLO3 KO sperm show impaired Ca^2+^ influx through CatSper channels in capacitated conditions (Chávez et al., 2014; Balbach et al., 2020) indicating a functional relationship between these two channels. In mouse, CatSper channels are strongly activated by pH alkalinization (Ren et al., 2001; Kirichok et al., 2006). Therefore, it has been suggested that hyperpolarization mediated by SLO3 contributes to the activation of CatSper channels by increasing pH_i_ through the activation of sNHE (Wang et al., 2007; Chávez et al., 2014; Windler et al., 2018; de Prelle et al., 2022). This sNHE has a putative voltage sensor domain that could be activated by Em hyperpolarization. To investigate this possible association, WT and SLO3 KO sperm were loaded with the probes BCECF-AM and DiSC_3_(5) to simultaneously evaluate pH_i_ and Em during capacitation, respectively. As shown in Figure 2F, WT sperm were observed in two well-defined populations: cells that showed high pH_i_ and low pH_i_ (higher or lower BCECF fluorescence, respectively). Notably, the population with higher pH_i_ also displayed hyperpolarized Em (evidenced by a higher DiSC_3_(5) fluorescence) while the subset with more acidic cytoplasm presented a depolarized Em (Figure 2E, upper panel). This result suggests that the population that has higher pH is hyperpolarized because alkaline pH activates SLO3 resulting in hyperpolarized Em.

To further explore this, the same experiment was performed in SLO3 KO sperm. As shown in Figure 2F, sperm from SLO3 KO mice also distributed in two well-defined populations, one with a more alkaline pH_i_ and another one with a more acidic pH_i._ Interestingly, both of them presented a depolarized Em (Figure 2E, lower panel), indicating that alkalization of pH_i_ might occur even in the absence of Em hyperpolarization. As controls, valinomycin and KCl were used to modulate sperm cells Em state. Valinomycin induced an increase in hyperpolarized cells (which present alkaline pH_i_) and KCl induced a depolarization of the Em. These results support that Em hyperpolarization depends on alkalization but that SLO3 is not driving pH_i_ alkalinization since this phenomenon may occur in the absence of Em hyperpolarization.

Collectively, these findings indicate that an increase in intracellular pH takes place regardless of variations in membrane potential. Despite exhibiting intracellular alkalinization associated with capacitation, sperm from SLO3 KO mice demonstrate compromised hyperactivated motility under *in vitro* conditions.

### Sperm from SLO3 KO mouse can migrate through the oviduct and reach the *ampulla*

The *in vitro* results showing impaired HA in SLO3 KO sperm led us to anticipate that sperm lacking SLO3 channels would be unable to ascend to the fertilization site. To evaluate the ability of SLO3 KO sperm to migrate in the female tract, two different mating schemes were performed. Super-ovulated WT females were mated with EGFP/DsRed2-SLO3 WT or KO male mice for 40 min either: 1) 12 h after hCG injection (AM mating) or; 2) immediately after hCG injection and left overnight (ON mating). Copulatory plugs were checked 13 h post hCG injection and, in every case, the presence of sperm in the female oviduct was verified by epifluorescence microscopy. We found that only 33.3% and 50% of the AM and ON matings were successful when using KO mice, respectively. In contrast, 80% of AM and 100% of ON schemes with WT males resulted in a successful mating (Supplementary Figure S1). Thus, KO males mated fewer times in the restricted mating schemes than WT male mice.

**FIGURE 3:**
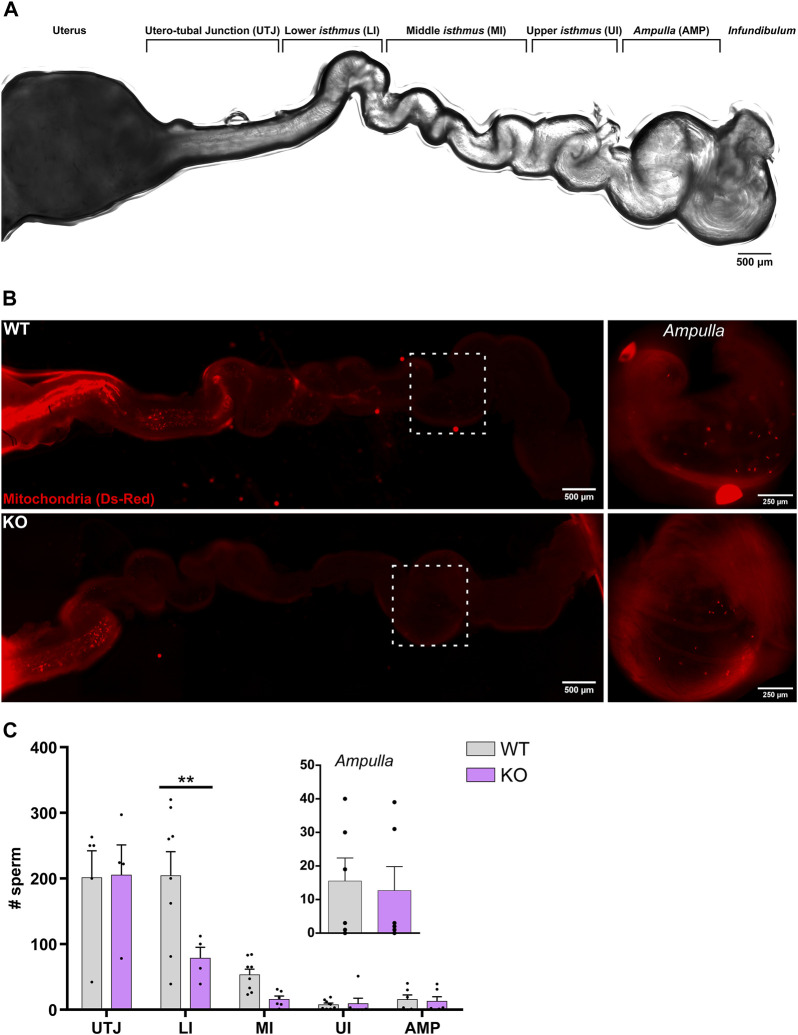
Evaluation of sperm migration *in vivo* after AM mating. WT or KO male mice and super-ovulated WT females were mated. Successful mating was evidenced by the presence of vaginal plug. Mice were mated at 5AM and plugs were checked 35 min later (AM). **(A)** Representative bright field image of the female reproductive tract depicting the different regions. **(B)** Representative epifluorescence images of DsRed2 fluorescence visible through the female tract 4 h post-coitus. Upper-left panel shows sperm from a WT mouse through the entire oviduct and the upper-right panel shows a zoom-in of the *ampulla* region. Lower-left panel shows sperm from a KO mouse through the entire oviduct and the lower-right panel shows a zoom-in of the *ampulla* region, showing the presence of sperm from KO mice in the region. **(C)** Quantification of the number of sperm in WT or KO mice at the different oviduct regions, utero-tubal junction (UTJ), lower *isthmus* (LI), mid *isthmus* (MI), upper *isthmus* (UI) and *ampulla* (AMP). Each dot represents the count for one oviduct. Values represent the mean ± SEM of at least three different experiments (N ≥ 3; 4 WT and 3 KO males, 7 females). **p < 0.01 stands for statistical difference between WT and KO. Multiple *t*-test corrected for multiple comparisons using the Holm-Sidak's method was performed.

Imaging of sperm transit through the oviduct was performed after mating super-ovulated WT females with either WT or KO male mice, to evaluate sperm migration. The number of sperm cells observed in the utero-tubal junction (UTJ), the *isthmus* and the *ampulla* were counted. For a more detailed description, the *isthmus* was arbitrary divided into three segments: lower (LI), mid (MI) and upper *isthmus* (UI) from closest to the UTJ towards closest to the *ampulla*, respectively (Figure 3A). Observation after 16 h of hCG injection (AM mating), revealed that sperm from WT males were present in each segment of the oviduct as previously described (Hino et al., 2016; La Spina et al., 2016; Muro et al., 2016). The highest number of sperm was observed in the UTJ (∼200 sperm cells) but it gradually decreased towards the *ampulla*. In this last segment, very few sperm cells were observed (La Spina et al., 2016). Reduced number of KO sperm were found in the LI, the sperm reservoir, compared to controls (∼200 and 78 sperm cells from WT and KO, respectively), (Figure 3C).

Surprisingly, although KO sperm have impaired HA motility *in vitro*, they were found in every segment of the oviduct (Figures 3B, C). Remarkably, sperm from KO males were found in the *ampulla*, the site of fertilization, in similar number as observed for WT sperm (∼13 sperm cells) (Figures 3A, B). A similar trend was observed when females were mated overnight (ON mating) with WT or KO male mice (Supplementary Figures S2A, B). These results indicate that Em hyperpolarization driven by SLO3 channels is not essential for sperm migration in the oviduct, suggesting that other factors independently of SLO3 might activate mouse sperm CatSper channels and, thus, HA in the mouse female genital tract. Alternatively, the limited levels of hyperactivation observed *in vitro* may be adequate for sperm migration within the oviduct.

### Most sperm from SLO3 KO mice arrive at the site of fertilization with an intact acrosome

**FIGURE 4:**
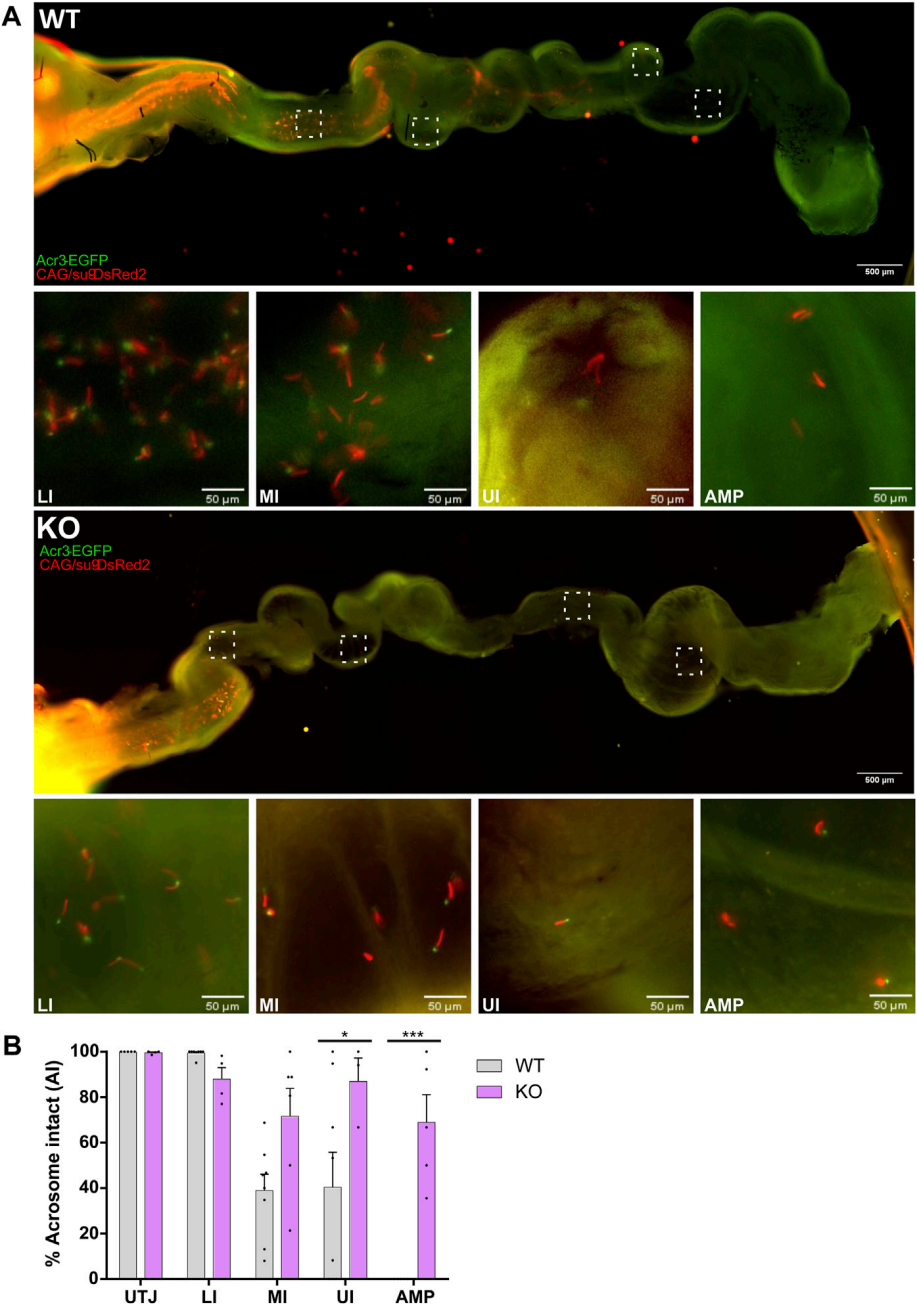
AE in the female oviduct after AM mating. Super-ovulated WT females were mated with EGFP/DsRed2-SLO3 WT or KO male mice. The presence of vaginal plug and sperm in the oviduct was used as an indicator of successful mating. Mice were mated at 5 AM and plugs were checked 40 min later (AM mating). **(A)** Representative epifluorescence images of DsRed2 and EGFP fluorescence through the female tract 4 h post-coitus. Upper panels show sperm from WT mouse through the entire oviduct, insets of the different segments of the oviduct are displayed as boxes and images acquired with higher magnification of the four different regions, lower *isthmus* (LI), mid *isthmus* (MI), upper *isthmus* (UI) and *ampulla* (AMP) are shown. Lower panels show the transit of sperm from EGFP/DsRed2-SLO3 KO mice through the oviduct. **(B)** Quantification of the percentage of acrosome intact (AI) sperm of WT or KO mice for each oviduct in its different regions, utero-tubal junction (UTJ), LI, MI, UI and AMP. Each dot represents the count for one oviduct Values represent the mean ± SEM of at least three different experiments (N ≥ 3; 4 WT and 3 KO males, 7 females). ***p < 0.001, *p < 0.05 stands for statistical difference between WT and KO. Multiple t-test corrected for multiple comparisons using the Holm-Sidak’s method was performed.

The acrosomal status of sperm was evaluated in each segment of the oviduct. The presence of EGFP fluorescence indicates a sperm with intact acrosome, while absence of EGFP denotes sperm that have undergone AE. In agreement with previous observations of our group (La Spina et al., 2016), sperm from WT mice began AE in the MI and UI, and no acrosome intact sperm were found in the *ampulla*, when the AM mating was performed (Figure 4A upper panel B). When mating occurs ON, the percentage of acrosome intact sperm was comparable to the AM mating at the UTJ but decreased in each segment of the isthmus (Supplementary Figures S2A, C). Only 2 out of a total of 48 sperm that reached the *ampulla* were acrosome intact when five different ampullas were analyzed in the ON matings.

Imaging of sperm from KO mice revealed that, similar to WT, most sperm in the UTJ and lower *isthmus* were predominantly acrosome intact. However, the most striking differences between both genotypes were that sperm from KO males reaching the *ampulla* were mostly acrosome intact (69% and 84% of sperm for the AM and ON mating, respectively). The percentage of acrosome intact sperm from WT mice in the *ampulla* was 0% and 4% for the AM and ON matings, respectively (Figure 4B; Supplementary Figure S2C). All together, these results indicate that: 1) the occurrence of AE is not required for sperm migration through the female tract; 2) Em hyperpolarization driven by SLO3 channels is essential for the ability of mouse sperm to undergo AE.

### Sperm from SLO3 KO mice do not fertilize *in vivo*

**FIGURE 5:**
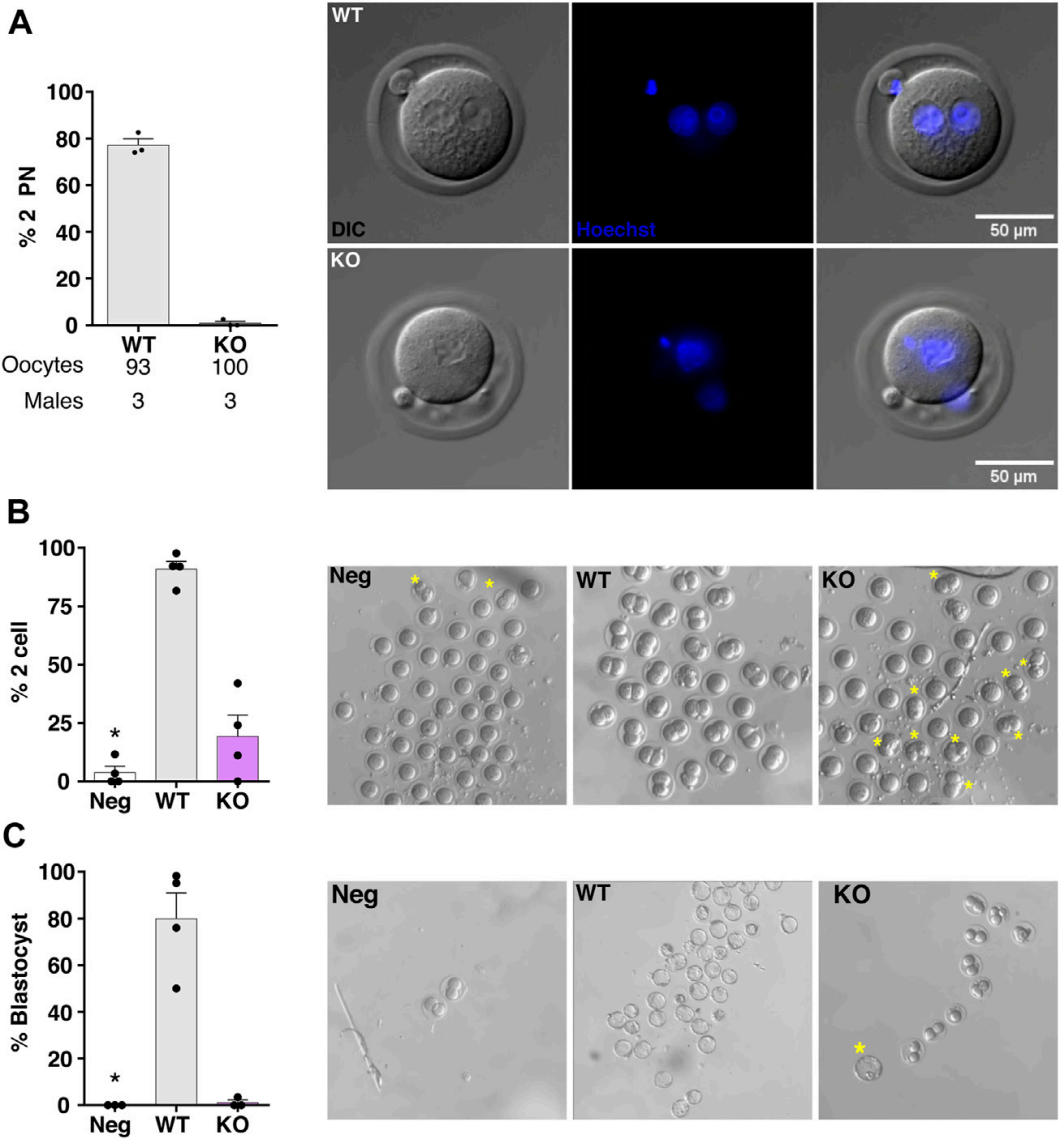
*In vivo* fertilization and embryo development in SLO3 KO sperm. COCs were collected 20 h after hCG injection from super-ovulated WT females mated ON with EGFP/DsRed2-SLO3 WT or KO male mice or not mated as a negative control (Neg). The presence of a vaginal plug was used as an indicator of successful mating. **(A)** Presence of 2-PN was assessed by epifluorescence imaging of Hoechst. The percentage of 2-PN was plotted for the WT and KO matings for three independent experiments (*N* = 3; 3 WT and 3 KO males, 6 females), and representative images are shown on the right, upper panel displays 1-cell embryo obtained from mating with WT males and lower panel with KO males. Non-parametric Mann-Whitney test was performed to evaluate differences between WT and KO. **(B,C)** A total of 16 different females were mated with either WT, KO or not mated (*N* = 4, 3 WT and 3 KO males). **(B)** The percentage of 2-cell embryos was plotted for each mating and the panel on the right shows representative images of 1-cell development to 2-cell embryos, of four independent experiments using three different pairs of KO and WT males, yellow asterisks indicate the presence of 2-cell embryos in the KO and Neg control. **(C)** The percentage of blastocyst for each mating was plotted and representative images for each genotype are shown, yellow asterisks represent presence of blastocyst in the KO mating. *p < 0.05 stands for statistical difference between WT and KO. One-way ANOVA in combination with Dunnet’s multiple comparisons test was performed against the control condition (WT).

Despite that the great majority of SLO3 KO sperm that reach the *ampulla* remain acrosome intact, a considerable proportion of them (∼20%) arrive at the site of fertilization acrosome reacted. In WT mice, acrosome reacted sperm can penetrate the *zona pellucida* (ZP) and fuse with the oocytes (Inoue et al., 2011). To investigate if acrosome reacted sperm from SLO3 KO mice can fertilize the ovulated MII-oocytes, super-ovulated WT females were mated ON with WT or SLO3 KO males. A set of super-ovulated females that were not mated were used as a negative control. The potential one-cell (1-cell) embryos were collected from the *ampulla* 20 h post hCG injection (Buffone et al., 2009b) and incubated *in vitro* for additional 22 h to reach the 2-cell stage. As expected, only 3.75% of oocytes reached the 2-cell stage in the negative control whereas 90.8% of the oocytes obtained from females mated with WT male mice developed to 2-cell embryos. Interestingly, 19.3% of oocytes from females mated with SLO3 KO male mice achieved 2-cell stage *in vitro* (Figure 5B). Although no significant difference was found in the percentage of 2-cell embryos between the negative control and the SLO3 KO, 2-cell embryos were transferred to a medium that supports blastocyst development. Almost 80% of the 2-cell embryos obtained from females mated with WT males develop to blastocyst, while only 1 out of 52 2-cell embryos developed to blastocyst when females were mated with SLO3 KO males (Figure 5C).

To investigate if the proportions of 2-cell embryos (∼20%) corresponding to females mated with SLO3 KO males were the result of fertilization by this small subset of acrosome-reacted sperm that reached the *ampulla*, formation of 2-PN was evaluated. In this case, the potential 1-cell embryos collected *in vivo* were fixed and stained with Hoechst. Only 1 out of 100 oocytes presented 2-PN when WT females were mated with SLO3 KO males (Supplementary Figure S3). In contrast, WT sperm produced ∼80% of 1-cell embryos with 2-PN (Figure 5A), indicating that previous observations were due to parthenogenesis and not to actual fertilization.

### SLO3 KO sperm fail to penetrate the ZP and fuse with the oocytes *in vitro*

**FIGURE 6:**
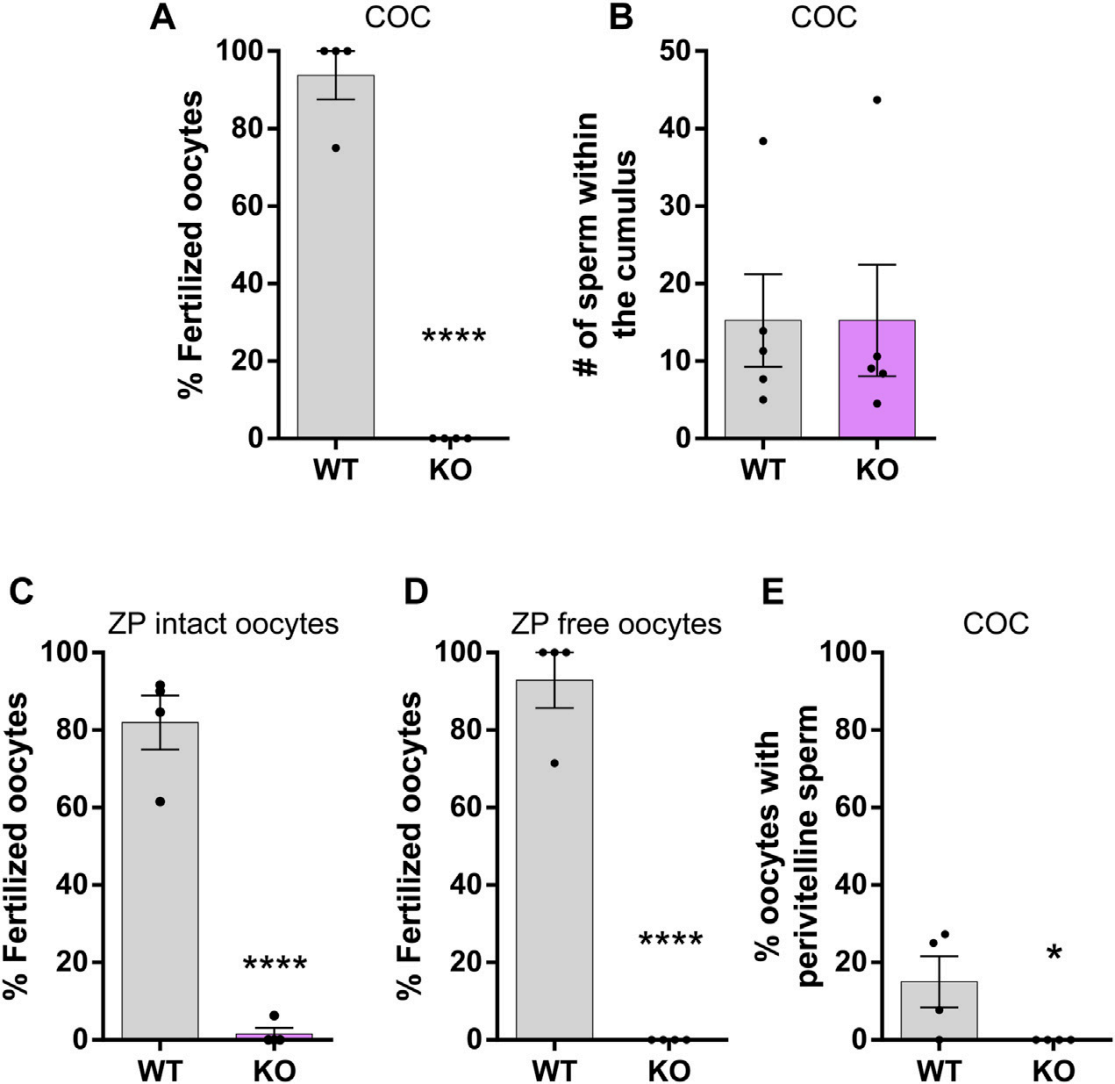
*In vitro* fertilizing ability of SLO3 KO sperm. **(A)** Capacitated epididymal sperm from SLO3 KO or WT mice were used to inseminate cumulus-intact oocytes (COC) (*n* = 4, 49 and 59 oocytes analyzed for WT and KO, respectively) and the percentage of fertilization was determined. Oocytes were considered fertilized when at least one decondensing sperm nucleus or two pronuclei were observed in the oocyte cytoplasm. **(B)** Capacitated epididymal sperm from SLO3 KO or WT mice previously exposed to Hoechst 33342 were co-incubated with COC for 15 min and the number of stained sperm within the cumulus was determined (*n* = 5, 89 and 85 oocytes analyzed for WT and KO, respectively). Capacitated epididymal sperm from SLO3 KO or WT mice were used to inseminate ZP-intact oocytes **(C)** (*n* = 4, 48 and 54 oocytes analyzed for WT and KO, respectively) or ZP-free oocytes **(D)** (*n* = 4, 53 and 53 oocytes analyzed for WT and KO, respectively) and the percentage of fertilization was determined. As in **(A)** oocytes were considered fertilized when at least one decondensing sperm nucleus or two pronuclei were observed in the oocyte cytoplasm. **(E)** Percentage of oocytes showing perivitelline sperm in **(A)**. In all cases “*n*” means the number of males analyzed per assay and matches with the number of independent experiments. Results represent the mean ± SEM. *p < 0.05 **p < 0.01, ***p < 0.001. T-student test was performed.

As an approach to investigate the reasons for the lack of *in vivo* fertilization, each of the stages of fertilization was analyzed *in vitro*. First, cumulus oocytes complexes (COC) were inseminated with WT or SLO3 KO sperm and the percentage of fertilized oocytes analyzed 3 h later. Results showed that mutant sperm were completely unable to fertilize COC (Figure 6A) as previously observed *in vivo*. To examine whether this result was due to a defect in the sperm ability to penetrate the cumulus oophorus that surrounds the oocytes, sperm were stained with Hoechst 33342 prior to insemination of COC and the number of fluorescent sperm within the cumulus mass analyzed 15 min later. No differences between groups (Figure 6B) were observed, supporting the existence of sperm functional deficiencies in subsequent stages of the fertilization process. To analyze the ability of mutant sperm to penetrate the ZP, ZP-intact oocytes devoid of *cumulus* cells were inseminated with control or mutant sperm and the percentage of fertilized oocytes analyzed 3 h later. Under these conditions, SLO3 KO sperm were completely unable to fertilize the oocytes (Figure 6C), indicating a clear defect of mutant sperm to penetrate the ZP. Whereas this observation could explain the lack of fertilization of COC both *in vitro* and *in vivo*, the possibility exists that SLO3 KO sperm also exhibit deficiencies to fuse with the oolemma that could not be detected due to deficiencies in the stage that precedes gamete fusion, i.e., ZP penetration. In view of this, the fusion ability of SLO3 KO sperm was analyzed using ZP-free oocytes and the percentage of fertilized oocytes analyzed 1 h later. The observation that mutant sperm were still unable to fertilize ZP-free oocytes (Figure 6D) indicated clear defects in the ability of mutant sperm to fuse with the oolemma. This result led us to reanalyze the presence of perivitelline sperm in oocytes from COC inseminated with either WT or mutant sperm, observing that whereas several oocytes exposed to WT suspensions exhibited sperm in the perivitelline space (Figure 6E), consistent with the block of polyspermy triggered by gamete fusion, none of the oocytes co-incubated with the mutant cells showed perivitelline sperm. Altogether, these observations support serious defects of SLO3 KO sperm at both sperm-ZP interaction and gamete fusion as responsible for the lack of fertilization of COC observed under *in vitro* and *in vivo* conditions.

## Discussion

Em hyperpolarization that occurs during capacitation is essential for fertilization (Santi et al., 2010; Zeng et al., 2011; Chávez et al., 2014; Puga Molina L. C. et al., 2018). In mice and humans, hyperpolarization modulates Ca^2+^ signaling, required for AE (De La Vega-Beltran et al., 2012; Torrezan-Nitao et al., 2020; Balestrini et al., 2021) and is necessary and sufficient to prepare sperm cells to undergo this special type of exocytosis (Santi et al., 2010; De La Vega-Beltran et al., 2012; Balestrini et al., 2021). Em hyperpolarization primes human sperm cells to undergo AE, by decreasing the [Ca^2+^]_i_ oscillations that prevent initiation of AE (Balestrini et al., 2021). It has been previously shown that SLO3 activation and Em hyperpolarization is important for Ca^2+^ entry though CatSper channels (Chávez et al., 2014; Balbach et al., 2020) indicating a functional relationship between these two channels and a possible role of SLO3 in HA motility. Because most of the studies were performed *in vitro*, its relevance during *in vivo* capacitation within the female reproductive tract has yet to be elucidated.

It is well established that experiments performed *in vitro* might not necessary reflect what occurs in a physiological context. In this regard, almost every study using mice as a model to evaluate sperm capacitation are performed using cells obtained from the cauda of the epididymis. These cells, although mature, have not been exposed to seminal plasma, and therefore, they have not experienced changes in osmolality and intracellular pH and also lack exposure to the seminal proteins and other components that play specific roles in the female reproductive tract (Peitz and Olds-Clarke, 1986; Mcgraw et al., 2015). Additionally, these sperm cells are never exposed to the fluids and cells found in the female reproductive tract. Despite that capacitation is carried out in a defined medium that allows sperm to fertilize oocytes *in vitro*, the efficiency is considered very poor. For example, while *in vivo*, only a few sperm that arrive at the site of fertilization are necessary to fertilize most of the MII-oocytes, *in vitro* fertilization is performed in the presence of thousands of male gametes.

Thus, there is a great need to evaluate the molecular and cellular events that have been associated with capacitation in the physiological context. The literature is full of examples showing contradictory results when *in vivo* and *in vitro* conditions are compared. Proteins that were believed to participate in ZP binding or sperm-oocyte fusion by *in vitro* experimentation did not show a significant phenotype when they were knocked-out [see many examples in Okabe (2013), Mahé et al. (2021), Yanagimachi (2022)]. Protein tyrosine phosphorylation (pY) has been considered a hallmark of *in vitro* capacitation (Visconti et al., 1995). However, a recent report showed that sperm that reach the *ampulla* lack pY, questioning the relevance of pY during capacitation *in vivo* (Ded et al., 2020). Similarly, AE was thought to be induced by sperm binding to ZP (Saling et al., 1979), these conclusions where derived from *in vitro* experimentations where solubilized ZP was used (Buffone et al., 2009a; Ren and Xia, 2010). However, *in vivo* observations of sperm with EGFP in their acrosomes demonstrated that sperm undergo AE prior contacting the ZP in the upper segments of the oviduct (Hino et al., 2016; La Spina et al., 2016; Muro et al., 2016).

Thus, to investigate the role of sperm Em hyperpolarization *in vivo*, a new transgenic mouse was used by crossing EGFP/DsRed2 sperm with SLO3 KO mice. These mice were used to evaluate the role of sperm Em within the oviduct with two main outcomes: 1) Sperm migration by evaluating DsRed2 fluorescence; 2) incidence of AE *in vivo* by evaluating the presence of EGFP. Compared to WT sperm, EGFP/DsRed2-SLO3 KO mice were similar in sperm concentration but presented a reduced tail morphology and progressive motility. They also cannot undergo AE or develop HA *in vitro* in agreement with previous reports on SLO3 KO mice (Santi et al., 2010; Zeng et al., 2011).

For migrating through the female reproductive tract, sperm requires HA (Ho et al., 2009). Also, peristaltic movements of the oviduct play a role in sperm ascend to the *ampulla* (Fujihara et al., 2018). Moreover, it has been shown that transport to the *ampulla* was fastest during the peri-ovulatory period (Hino and Yanagimachi, 2019). Other mechanisms have been proposed to be necessary for sperm guidance such us thermotaxis (Bahat et al., 2003; De Toni et al., 2016), chemotaxis (Gatica et al., 2013), and rheotaxis (Miki and Clapham, 2013). This last mechanism has recently been challenged by observations indicating that the flow in the female tract goes from the uterus to the *ampulla* (Hino and Yanagimachi, 2019; Bianchi et al., 2021). Although SLO3 KO sperm display reduced levels of HA *in vitro*, they are able to ascend through the UTJ and colonize all segments of the oviduct. The number of sperm colonizing the lower *isthmus* was found to be slightly reduced in oviducts from matings with SLO3 KO mice compared to WT consistent with a reduction of progressive motility observed *in vitro*. The lower *isthmus* is considered to be the sperm reservoir where sperm interact with the epithelium, and HA motility is required for sperm to detach and swim freely towards the *ampulla* (Suarez and Pacey, 2006; Suarez, 2016). This is in accordance to observations from Ppp3cc KO mice and Catsper1 KO mice, where sperm motility is impaired and therefore migration through the UTJ was reduced (Ho et al., 2009; Miyata et al., 2015; Ded et al., 2020). In these KO mice, fewer sperm where able to reach the *ampulla* when compared to WT indicating that not only oviduct flow and peristaltic contractions are necessary for migration but most importantly, the ability of sperm to HA. Our results suggest that albeit sperm count in the lower *isthmus* was found to be reduced in the EGFP/DsRed2-SLO3 KO, they still were able to colonize all segments of the oviduct similar to the WT.

Despite the absence of SLO3-dependent hyperpolarization, HA motility might not be severely impaired within the tract. In this regard, one possible explanation is that other factors besides SLO3 can activate CatSper in the female tract. One of the main activators of mouse CatSper channels is the increase in intracellular pH. One hypothesis is that hyperpolarization of Em activates the sperm specific NHE (sNHE) through its voltage-sensing domain (Windler et al., 2018), promoting the cytoplasmic alkalinization and CatSper channel activation and resulting in an increase in [Ca^2+^]_i_ required for HA motility (Hwang et al., 2019; de Prelle et al., 2022). Recent findings have shown that hyperpolarization triggered an increase in pH_i_ (Chávez et al., 2020; Hernández-Garduño et al., 2022). However, our experiments using pH sensitive probes showed that the pH of SLO3 KO sperm is comparable to WT making this hypothesis debatable. One possible explanation for this result is that in the absence of SLO3 channels, other mechanism induces the cytoplasmic pH alkalinization to activate CatSper channels. Among them, the entry of HCO_3_^−^ can occur via different cotransporters such as NBC and NKCC (Arcelay et al., 2008; Wertheimer et al., 2008; Puga Molina L. D. et al., 2018) or using other transporter associated with CFTR function (Chávez et al., 2012). It is also possible that carbonic anhydrases (Wandernoth et al., 2010; José et al., 2015) or other NHE’s (Chen et al., 2016) may also contribute to pH_i_ alkalinization. In any case, such compensatory mechanisms should be more important *in vivo* than *in vitro* to explain the discrepancies between *in vitro* and *in vivo* results. Regardless of the mechanism involved, SLO3 KO sperm need to develop HA to reach the *ampulla*. We hypothesized that certain component in the female tract may be able to induce or facilitate the development of HA motility independently of SLO3 activation and pH_i_ increases. For example, progesterone has been shown to be important for CatSper activation in humans but not in mice (Mannowetz et al., 2017). It is likely that other components present in the fluid may activate partially or completely the opening of CatSper required for HA.

The occurrence of AE *in vivo* was analyzed by visualizing the presence of EGFP in different sections of the oviduct. Surprisingly, it was observed that more than 80% of SLO3 KO sperm reach the *ampulla* with their acrosomes intact in sharp contrast to WT sperm where, virtually all of them arrive at the site of fertilization acrosome reacted. Thus, it is confirmed by the *in vivo* results that Em is important for preparing the cells to undergo AE. However, a significant number (∼20%) of sperm from SLO3 KO mice arrived acrosome reacted to the *ampulla*. There are at least two potential explanations for this observation. Firstly, it could be attributed to the spontaneous loss of the acrosomal cap, a phenomenon observed in both cell death and degenerative processes, as seen in *in vitro* experiments. The occurrence of spontaneous AE is also noted in SLO3 knockout sperm, regardless of whether they are incubated under capacitating conditions or in the absence of any stimulants triggering the process. Secondly, *in vivo* hyperpolarization of the Em might be partially offset by other mechanisms, such as CFTR and/or ENaC channels (Puga Molina et al., 2017; Puga Molina L. D. et al., 2018). Additionally, these findings unequivocally indicate that AE is not a necessary condition for sperm migration through the oviduct. Prior to these observations, the reason for sperm undergoing AE in the mid *isthmus*, located at considerable distance from the oocyte and its surrounding layers, remained unclear. Recently, using FER1l5 KO mice, Morohoshi and collaborators also observed the presence of acrosome intact sperm in the *ampulla* (Morohoshi et al., 2023). The reason why sperm undergo AE in the upper segments of the oviductal isthmus remains unknown.

It is very well established that only acrosome reacted sperm are able to penetrate the ZP and fertilize the oocytes (Jin et al., 2011). Since certain sperm reached the *ampulla* even in the absence of the acrosomal cap, we assessed whether these gametes were capable of fertilizing the oocyte. However, when SLO3 KO male mice were mated with WT females no formation of 2-PN was observed, indicating that the absence of SLO3 prevented sperm from fertilizing the oocytes. To analyze the mechanisms underlying the lack of fertilization observed in the ampulla of females mated with SLO3 KO males, *in vitro* fertilization experiments were carried out using oocytes with or without their vestments. Of note, whereas the ability of SLO3 KO sperm to fertilize the oocytes *in vitro* had been previously analyzed (Santi et al., 2010; Zeng et al., 2011), these studies were incomplete as they did not evaluate fertilization of the complete cumulus oocyte complex (COC) which the sperm face in the oviduct, nor the penetration of the individual coats that surround the oocyte. i.e., cumulus oophorus and ZP, nor the presence of sperm in the perivitelline space as an indication of defects only in the gamete fusion step. Moreover, fertilization in those studies was evaluated as the percentage of cells reaching the two-cell stage, not allowing to exclude potential involvement of Slo3 in the subsequent embryo development process. The finding that, as previously observed *in vivo*, sperm from KO males could not fertilize the COC but could penetrate the cumulus mass similarly to WT sperm supported the existence of defects in subsequent stages of gamete interaction, i.e., ZP penetration and/or gamete fusion. This conclusion was confirmed by the observation that sperm from KO males failed to penetrate both ZP-intact and ZP-free oocytes, revealing clear defects in the ability of SLO3 KO sperm to both penetrate the resilient ZP and fuse with the oolema. In this regard, the finding that none of the oocytes from COC inseminated with mutant sperm exhibited sperm in the perivitelline space indicates that the lack of fertilization of COC *in vitro* is mainly due to defects in the stage of ZP penetration that precedes gamete fusion. The observation of perivitelline sperm in those COC inseminated with WT sperm, on the other hand, confirms the activation of the block to polyspermy known to be triggered by gamete fusion. Together, the *in vitro* fertilization results support the idea that the lack of fertilized oocytes in the ampulla of females mated with SLO3 KO males could be due to deficiencies of mutant sperm to penetrate the ZP and/or fuse with the oolema. Moreover, as SLO3 KO sperm exhibit defects to undergo the AR in the oviduct, it is likely that fertilization failure in the ampulla is mainly due to the observed defects in the occurrence of the AR known to be essential for both ZP penetration and gamete fusion. In this regard, whereas hyperactivation is also crucial for ZP penetration, the finding that mutant sperm could reach the ampulla favors the development of this vigorous motility and support AR defects as the principal responsible for fertilization failure within the oviduct.

Ultimately, developing a method to restore sperm membrane hyperpolarization could prove valuable in addressing certain cases of normospermic male infertility. This is supported by previous studies indicating that Em hyperpolarization, linked to human sperm capacitation, aligns with higher *in vitro* fertilization success rates (Baro Graf et al., 2020; Puga Molina et al., 2020).

## Materials and methods

### Reagents

Chemicals were purchased from the following sources: Bovine Serum Albumin (BSA) A7906, progesterone (Prog), Ca^2+^ ionophore A23187, Hyaluronidase, paraformaldehyde (PFA) and mineral oil were obtained from Sigma (St. Louis, MO, United States); pregnant mare serum gonadotropin (PMSG) and human chorionic gonadotropin (hCG) were purchased from Syntex (Bs. As., Argentina); Hoechst 33342 from Thermo Fisher Scientific (Waltham, MA, United States); valinomycin (Val) from Cayman Chemicals, (Ann Arbor, MI, United States); propidium iodide (PI) from Santa Cruz (Santa Cruz, CA, United States); and Vectashield from Vector (Newark, CA, United States). Chemicals were dissolved as follows: valinomycin, progesterone, A23187 were diluted in DMSO; Hoechst, PI and PFA in distilled water; PMSG and hCG in physiological saline solution and Hyaluronidase in PBS.

### Animals

C57BL/6 male mice expressing EGFP in the acrosome and DsRed2 in the mitochondria (EGFP/DsRed2 aka RGM) (Hasuwa et al., 2010), were mated with C57BL/6 female SLO3 KO mice (Santi et al., 2010). The resulting offspring EGFP/DsRed2 knock-out (KO), EGFP/DsRed2 Heterozygotes (HET) or EGFP/DsRed2 wild-type (WT) for SLO3 were used for the experiments. Superovulated 8-week-old F1 WT females (Hybrid F1: C57BL/6 male x BALB/c female) were used. Mice were housed in a room with controlled temperature (23°C), with light/dark cycles of 12 h (lights on from 07:00 a.m. to 07:00 p.m.), and *ad libitum* access to tap water and laboratory chow. Institutional animal care guidelines reviewed and approved by the Ethical Committees of the *Instituto de Biología y Medicina Experimental*, *Buenos Aires*, *Argentina* #32/2021, were followed in all experimental procedures. The Guide for Care and Use of Laboratory Animals approved by the National Institutes of Health (NIH) was strictly met.

### Sperm and embryo development medium

A modified Toyoda-Yokoyama-Hosi (modified TYH) medium was used (Toyoda et al., 1971) for sperm collection and manipulation. TYH containing 119.3 mM NaCl, 4.7 mM KCl, 1.71 mM CaCl_2_^.^2H_2_O, 1.2 mM KH_2_PO_4_, 1.2 mM MgSO_4_^.^7H_2_O, 0.51 mM sodium pyruvate, 5.56 mM glucose, 20 mM HEPES, and 10 μg/mL of gentamicin was used as non-capacitating medium (NC medium), while 5 mg/mL of BSA and 15 mM of NaHCO_3_ were added for capacitating medium (CAP medium). Medium pH was adjusted to 7.4 with NaOH.

One-cell embryos were cultured to two-cell embryos in TYH without 20 mM HEPES and in the presence of 4 mg/mL BSA and 25 mM NaHCO_3_ (TYH IVF medium). Development to blastocyst stage was performed in KSOM medium, containing 95 mM NaCl, 2.55 mM KCl, 1.7 mM CaCl_2_^.^2H_2_O, 0.37 mM KH_2_PO_4_, 0.20 mM MgSO_4_^.^7H_2_O, 0.18 mM sodium pyruvate, 0.22 mM glucose, 10 mM sodium lactate, 0.014 mM EDTA, 1 mM L-glutamine, 0.18 mg/mL penicillin G, 0.05 mg/mL streptomycin, 1 mg/mL BSA and 25 mM NaHCO_3_. Before use, TYH IVF and KSOM media were left in a 5% CO_2_ incubator overnight to reach pH = 7.4.

### Sperm collection and capacitation

After euthanizing the mice, we collected the cauda epididymis for sperm retrieval. The collected tissue was minced and then placed in 500 μL of TYH NC medium. Subsequently, we allowed the sperm cells to swim out (SO) of the cauda into the medium for 15 min at 37°C. Afterward, the sperm suspension was collected, and the concentration calculated using a Neubauer chamber. Sample were re-suspended to achieve a final concentration of 1 × 10^7^ cells/mL in the appropriate medium. Sperm morphology was examined in wet preparations under a microscope at ×400. The percentage of sperm cells with a normal and abnormal morphology was calculated. A morphological disorder was defined as any abnormality in sperm appearance or the head, tail, and remaining cytoplasmic residues. The average numbers of spermatozoa with normal morphology were calculated as percentages and at least 100 spermatozoa were analyzed per mouse. All slides were scored by the same individual. To achieve sperm capacitation, we incubated the sperm in TYH CAP medium for 60 min at 37°C. In order to induce pharmacological hyperpolarization or depolarization of the Em, sperm were capacitated in the presence of 1 μM valinomycin without (hyperpolarization condition) or with 40 mM KCl (depolarization condition).

### Determination of acrosomal exocytosis (AE) *in vitro* by flow cytometry

To evaluate AE *in vitro*, sperm EGFP/DsRed2 containing EGFP in the acrosome and DsRed2 in the mitochondria were capacitated with or without 1 μM Val and then incubated in the presence of 30 μM Prog, 10 μM A23187 or DMSO (vehicle) for 30 min at 37°C, as previously described (Muro et al., 2012; Hirohashi et al., 2015; Romarowski et al., 2015). To assess sperm viability, cells were loaded with 2 ng/μL PI immediately prior flow cytometry acquisition. FACSCanto II TM cytometer (Biosciences; Becton, Dickinson and Company) was used to record data as single cellular events. To define sperm population, forward-scatter area (FSC-A) and side-scatter area (SSC-A) data were collected from 20,000 events per sample. Doublets were excluded from analysis by analyzing a two-dimensional dot plot FSC-A vs. forward-scatter height (FSC-H). EGFP and PI fluorescence were collected using the filter for Fluorescein isothiocyanate (FITC; 530/30) and Peridinin chlorophyll protein complex (PerCP; 670LP) respectively. EGFP positive cells are acrosome intact sperm whereas acrosome reacted sperm lack EGFP fluorescence. Data analysis was performed with FlowJo software (X 10.0.7r2).

To assess AE, 10 different experiments were conducted (*N* = 10), 7 WT males were evaluated in all conditions. 4 HET males were evaluated for: Prog 50 μM and 5 HET for the rest of conditions evaluated. 9 KO males were evaluated for Prog 50 μM and 10 KO males were evaluated in the rest of the conditions.

### Sperm motility analysis

Non-capacitated sperm cells were diluted with 2-fold capacitating medium (CAP 2x: 10 mg/mL BSA and 30 mM NaHCO_3_) to reach a concentration of 2.5 × 10^6^ cells/mL and to prevent a non-specific attachment to the slide surface. To allow sperm to swim freely, 10 μL of the cell suspension was seeded in a slide with an 18 mm × 18 mm coverslip and sperm motility parameters were immediately analyzed using the Sperm Class Analyzer^®^ system (SCA v.6.2.0.1.; Microptic SL, Barcelona, Spain). Temperature was maintained at 37°C using a temperature-controlled stage. At least 10 fields were acquired and a minimum of 200 cells were analyzed. Average path velocity (VAP), curvilinear velocity (VCL), straight-line velocity (VSL), linearity (LIN), amplitude of lateral head displacement (ALH), and straightness (STR) were measured. For HA, sperm were incubated during 60 min under capacitating conditions before assessing their motility. Hyperactivated sperm were considered when presenting VCL ≥ 271 μm/s, LIN < 50%, and ALH ≥ 3.5 μm. A total of four Paired experiments (*N* = 4) were conducted using four different WT and four different KO males.

### Determination of Em and pH_i_ by flow cytometry

To assess Em and pH_i_, capacitated sperm were incubated for 20 min with 0.5 µM of a pH sensitive probe, BCECF-AM in NC medium. The probe was then washed by a gentle centrifugation (4 min at 500 g), and sperm cells were suspended in NC medium containing 1 nM of the membrane-potential sensitive dye DiSC_3_(5). About 1 μg/mL PI was added 30 s prior collecting data to assess sperm viability. Data were recorded as individual cellular events using a MACSQuant Analyzer 16 cytometer (Miltenyi Biotec). Forward-scatter area (FSC-A) and side-scatter area (SSC-A) data were collected from 20,000 events per sample to define sperm population. In all cases, doublet exclusion was performed by analyzing the two-dimensional dot plot of FSC-A versus forward-scatter height (FSC-H). Doublets exhibit a higher signal width or area-to-height ratio compared with single cells (singlets). Events deviating from the diagonal are doublets. Negative stain for PI was selected for living cells using the filter for Peridinin chlorophyll PerCP (667/30). Cells that incorporated BCECF-AM probe were detected using the filter for Fluorescein isothiocyanate (FITC; 530/30). Positive cells for DiSC_3_(5) were detected using the filter for Allophycocyanine (APC; 667/30). The addition of 1 μM Valinomycin and 40 mM KCl after each measurement was used as a control of Em modulation during CAP conditions. Data were analyzed using FlowJo software (X 10.0.7r2). Five Paired experiments (*N* = 5) were conducted using five different WT and five different KO males.

### *In vivo* imaging of sperm migration

To evaluate migration and AE *in vivo*, WT F1 female mice were super-ovulated by intraperitoneal injection of 5 IU of PMSG and 5 IU of hCG 48 h apart. Females were housed with EGFP/DsRed2-SLO3 WT or KO male mice 12 h after hCG, for 40 min: AM mating scheme; or immediately after hCG injection for 15 h: overnight mating scheme. Mating was confirmed by plug visualization. In all cases, 16 h after hCG injection (4 h after ovulation) females were euthanized and the oviducts were dissected and stretched, washed with CAP medium and mounted onto a chamber for epifluorescence microscopy imaging, as described by La Spina et al. (2016). Humidity and temperature (37°C) were controlled with a microscope stage chamber (Tokai Hit, Japan). Imaging of sperm in the oviduct was done using an inverted epifluorescence microscope (Olympus IX83, Japan) with an attached digital camera (CMOS Hamamatsu Orca Flash 4.0) and the acquisition Software CellSens Dimensions (Olympus, Japan). Z-stacks collecting DsRed2 fluorescence from the sperm midpiece and EGFP fluorescence from sperm acrosome were performed every 10 µm in each region of the oviduct, using U-FGWA and U-FBWA filters respectively. A mosaic was also obtained by imaging of the whole oviduct in low magnification (×4). Images were then processed using ImageJ 1.53q (National Institute of Health, United States) for manual counting of acrosome-intact and acrosome-reacted sperm within each Z-stack.

For AM mating a total of seven different females were used and each female was mated with either WT or KO males (4 WT and 3 KO males were used), while for the ON mating eight different females were housed with either WT of KO males (4 WT and 4 KO males were evaluated). Each oviduct was considered as one replicate.

### Determination of pronuclei formation

To evidence the first stage of fertilization, the presence of two-pronuclei (2-PN) was analyzed. Super-ovulated WT females were mated with EGFP/DsRed2-SLO3 KO or WT male mice ON. The *ampullas* were collected approximately 20 h post hCG injection and *cumulus-*oocyte complexes (COCs) were treated with 12.5 mg/mL Hyaluronidase in TYH IVF medium for 30 s, or until oocytes were free of *cumulus* cells. Oocytes were quickly rinsed in TYH IVF medium, fixed with 4% of PFA for 30 min and washed with PBS-BSA (4 mg/mL BSA). Staining for 10 min with 0.1 mg/mL Hoechst was performed, followed by a washing step with PBS-BSA. Oocytes were carefully mounted with Vectashield. Images were taken with an inverted epifluorescence microscope, (Olympus IX83) with an attached digital camera CMOS Orca Flash 4.0 (Hamamatsu, Japan) and the acquisition Software CellSens Dimensions (Olympus, Japan). 2-PN were identified by Hoechst fluorescence, UV light, or Differential interference contrast (DIC) microscopy. Images were processed with ImageJ 1.53q (National Institute of Health, United States) and the percentage of 2-PN was obtained.

Total of six females were used. Each female was paired with 1 WT or KO male (3 WT and 3 KO males were used). 93 oocytes and a 100 oocytes were counted for females mated with WT and KO, respectively.

### Embryo development to blastocyst stage *in vitro*

COCs from mated females were placed in drops of TYH IVF medium immersed in mineral oil, 20 h post hCG. Cells were immediately washed 3 times, with TYH IVF medium, to rinse off the *cumulus* cells and left in an incubator with 5% CO_2_ and 37°C for 24 h. The next day, progression to the two-cell (2-cell) stage was evaluated. Embryos in the 2-cell stage were washed and incubated in drops of KSOM medium immersed in mineral oil. After 72 h, development to blastocyst was observed. The percentage of blastocyst was obtained. Intermediate blastocyst, fully developed blastocyst and hatched blastocyst were counted as positive blastocysts, over the total of 2-cell embryos that were left to develop. A total 6 males (3 WT and 3 KO) and 12 WT females were used. A total of 220, 203 and 153 oocytes were collected from matings with KO males, WT males or not mated respectively. A total of 52, 183 and 7 two-cell embryos and 1,176 and 0 blastocysts were obtained for matings with KO males, WT males or not mated, respectively.

### *In vitro* fertilization assays

Mouse sperm (7 WT and 7 KO males) were allowed to swim out by mincing the cauda epididymis in 300 μL of CAP medium (Giaccagli et al., 2021) supplemented with 0.3% (w/v) bovine serum albumin (BSA), pH: 7.3–7.5 (“swim-out”). After 10 min, aliquots of the suspension were added to 300 μL of capacitation medium to a final concentration of 5–10 × 10^6^ cells/mL. Sperm suspensions were then incubated for 90 min at 37°C in an atmosphere with 5% (v/v) CO_2_ in air.

Gamete interaction assays were carried out as previously reported (Curci et al., 2020). Briefly, 25 female mice were superovulated by an injection of eCG (5 UI, Zoetis, Buenos Aires, Argentina), followed by hCG (5 UI, Zoetis) 48 h later. *Cumulus* oocyte complexes (COC) were collected from the oviducts 13–14 h after hCG administration and pooled. When needed, cumulus cells were removed by incubating the COC in 0.3 mg/mL hyaluronidase (type IV) for 3–5 min. In some cases, the *zona pellucida* (ZP) was dissolved by treating the oocytes with acid Tyrode solution (pH 2.5) for 10–20 s (Nicolson et al., 1975). COC and ZP-intact oocytes were inseminated with a final concentration of 1–5 × 10^5^ cells/mL and gametes were co-incubated for 3 h at 37°C in an atmosphere of 5% (v/v) CO_2_ in air. ZP-free oocytes were inseminated with a final concentration of 1–5 × 10^4^ cells/mL and gametes co-incubated for 1 h under the same incubation conditions. In all cases, oocytes were recovered at the end of incubation, washed, fixed with 2% (w/v) paraformaldehyde in PBS, stained with 10 μg/mL Hoechst 33342 and the percentage of fertilization determined under epifluorescence microscope (×200). Oocytes were considered fertilized when at least one de-condensing sperm nucleus or two pronuclei were observed in the oocyte cytoplasm. Oocytes from COC fertilization studies were also analysed for presence sperm in the perivitelline space.

For the cumulus penetration assay (Ernesto et al., 2015), capacitated mouse sperm were incubated for 15 min in medium containing 0.01 μg/μL Hoechst 33342, and then washed with capacitating medium. COC were inseminated (final concentration 1–5 × 10^5^ cells/mL) with labelled sperm and, after 15 min, washed, fixed, and mounted on slides. The number of sperm present within the cumulus was determined by epifluorescence using plan 20× NA 0.50 objective lenses.

### Statistical analysis

Data are expressed as mean ± standard error of the mean (SEM) of at least three independent experiments for all determinations. Statistical analyses were performed using GraphPad Prism 6.01 software (La Jolla, CA, United States). Parametric or non-parametric comparisons were performed following the data distribution. Two-way analysis of variance (ANOVA) was performed to evaluate EA *in vitro*, CASA parameters, and EA *in vivo*; followed by a multiple comparison test. To evaluate the association between the mating success and genotype the chi^2^ test was used. *In vitro* fertilization studies were analysed by on-tail Student’s test. *p*-Values (p) under 0.05 (*p* < 0.05) were considered statistically significant.

## Significant statement

In this study, we illustrate the crucial role of plasma membrane hyperpolarization in sperm migration within the female reproductive tract. Sperm that do not undergo this hyperpolarization can reach the fertilization site but are unable to penetrate the zona pellucida, undergo acrosomal exocytosis, and fuse with the oocytes.

## Data Availability

The raw data supporting the conclusion of this article will be made available by the authors, without undue reservation.
