# Supplementary figures and images for: Membrane potential hyperpolarization: a critical factor in acrosomal exocytosis and fertilization in sperm within the female reproductive tract
Source: Front Cell Dev Biol. 2024 May 13;12:1386980. doi: 10.3389/fcell.2024.1386980 (PMC11128623; doi:10.3389/fcell.2024.1386980)

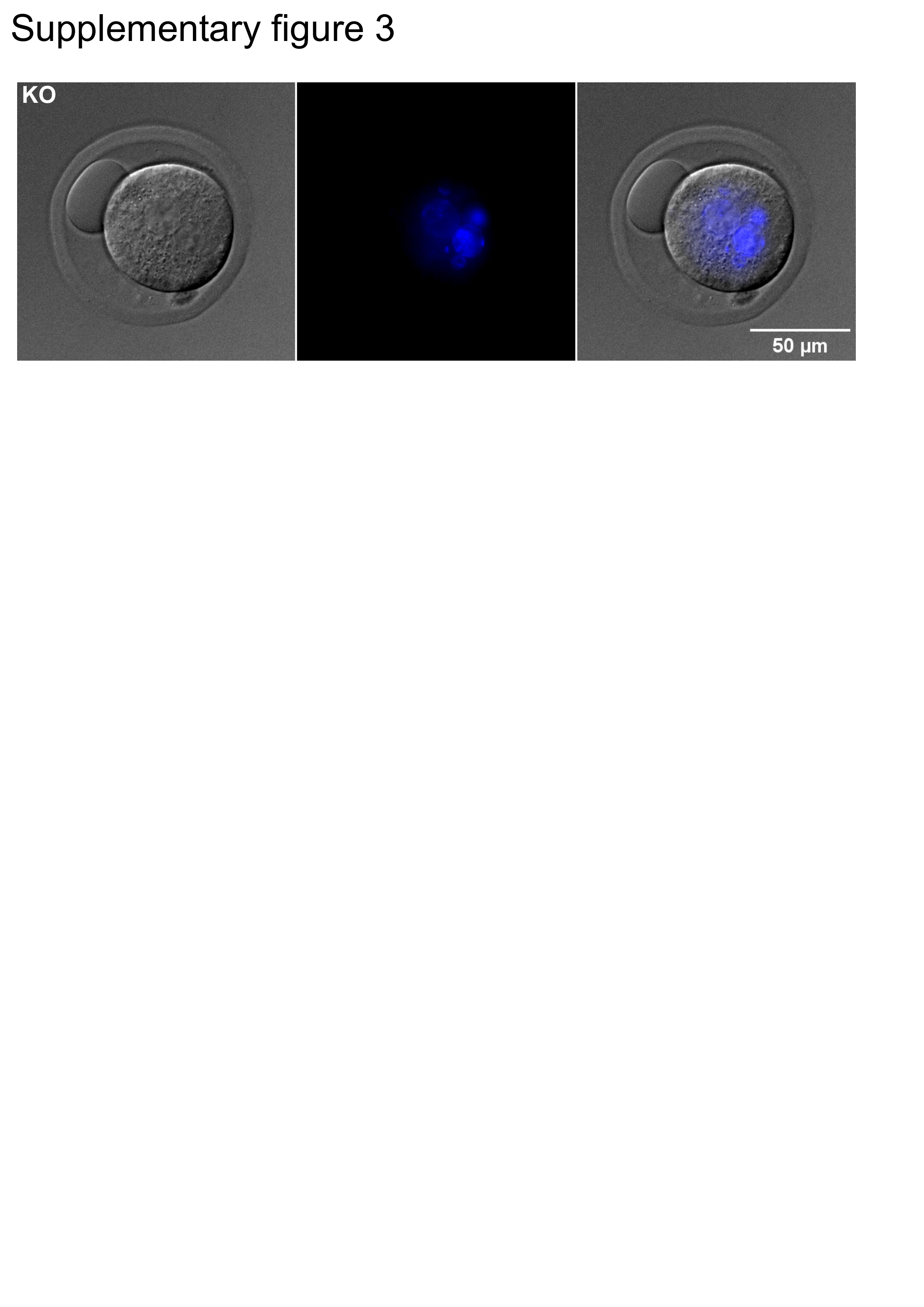

Supplement: Supplementary file 1 [file Image3.jpg]

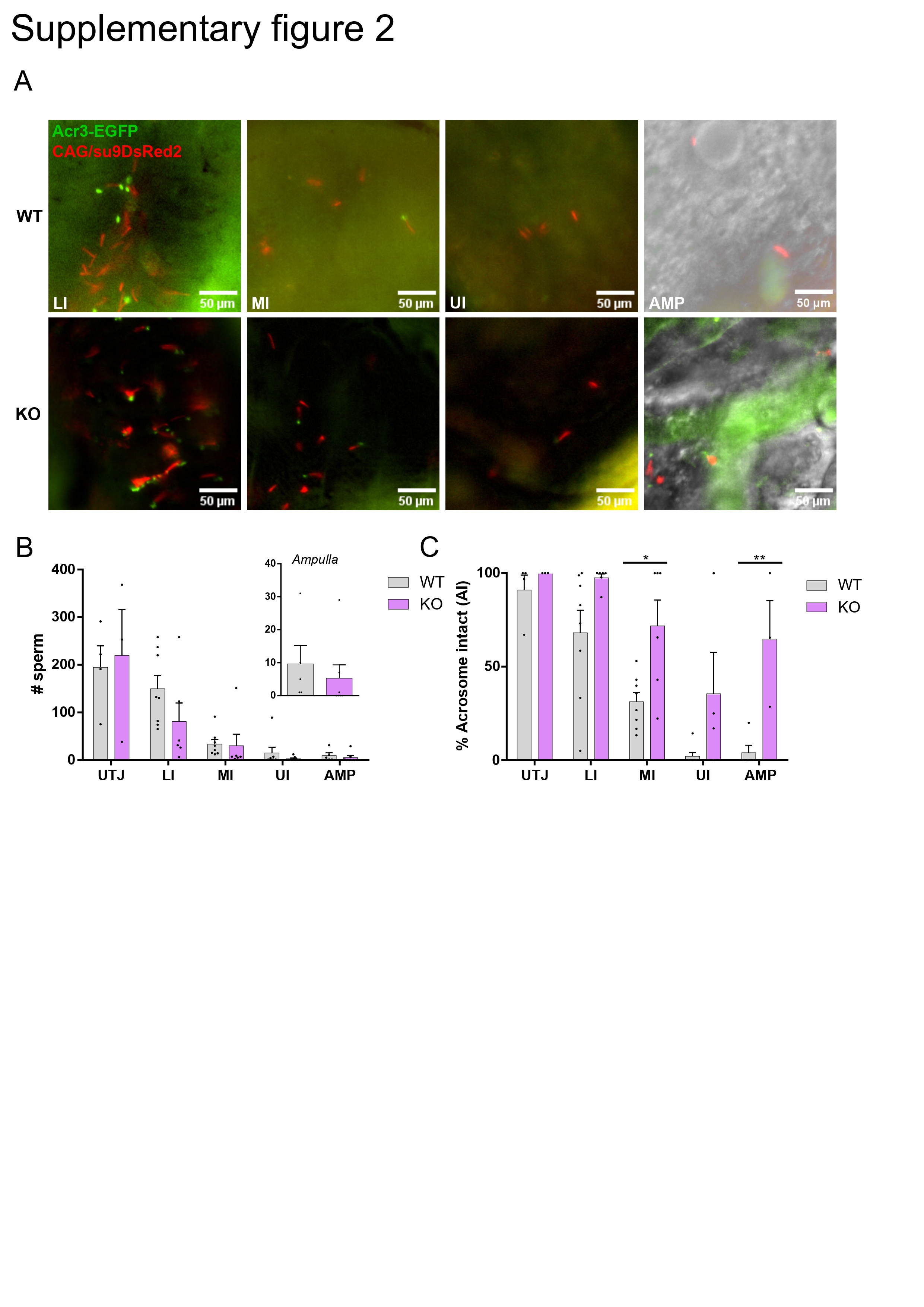

Supplement: Supplementary file 2 [file Image2.jpg]

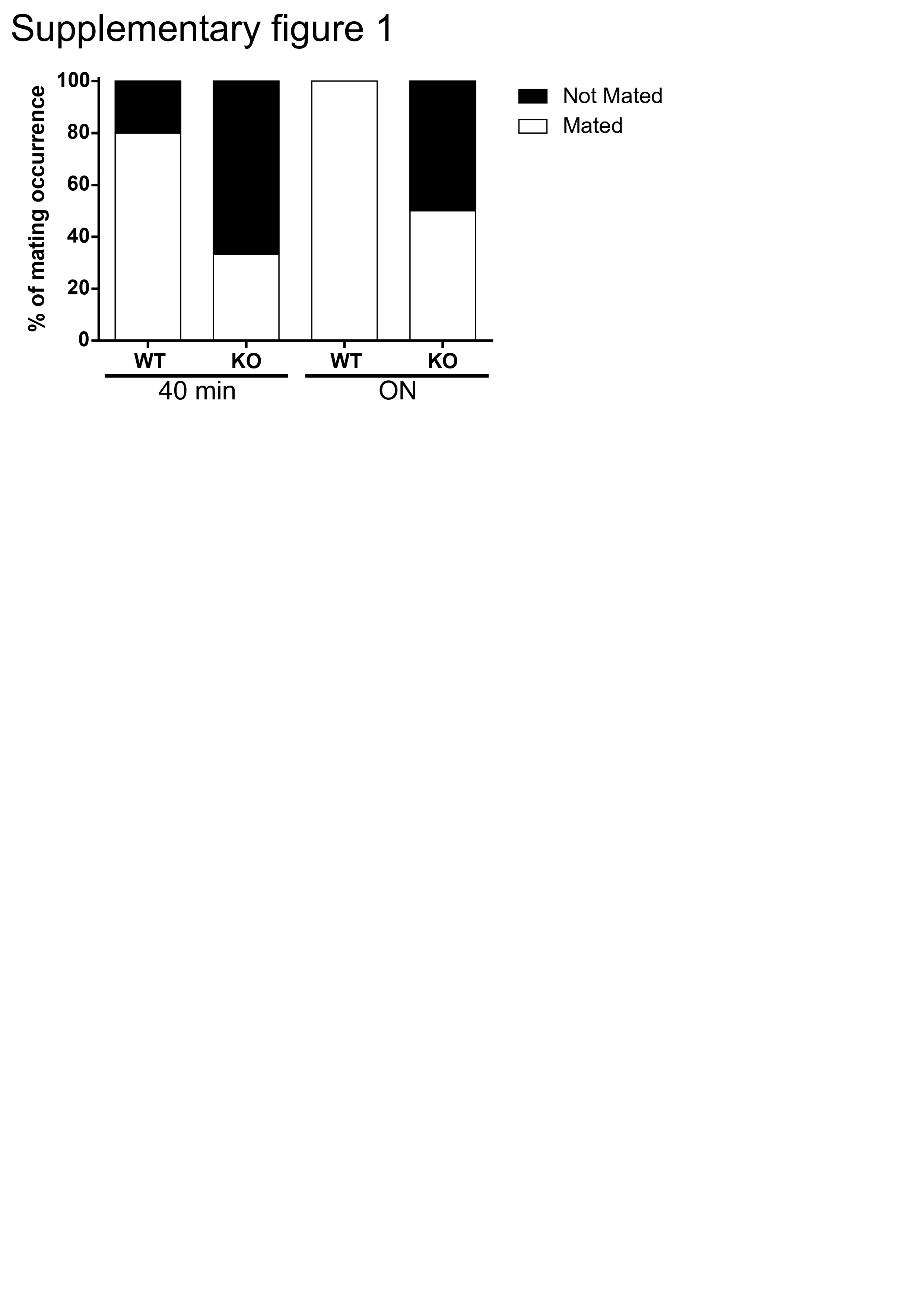

Supplement: Supplementary file 3 [file Image1.jpg]

Supplementary figure 1

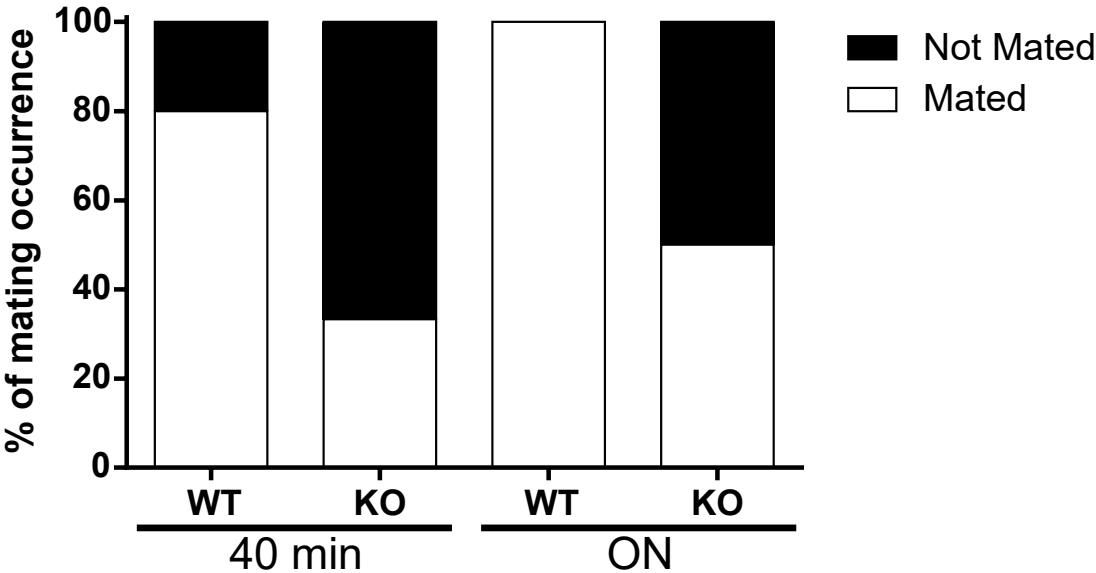

# Supplementary figure 2

A

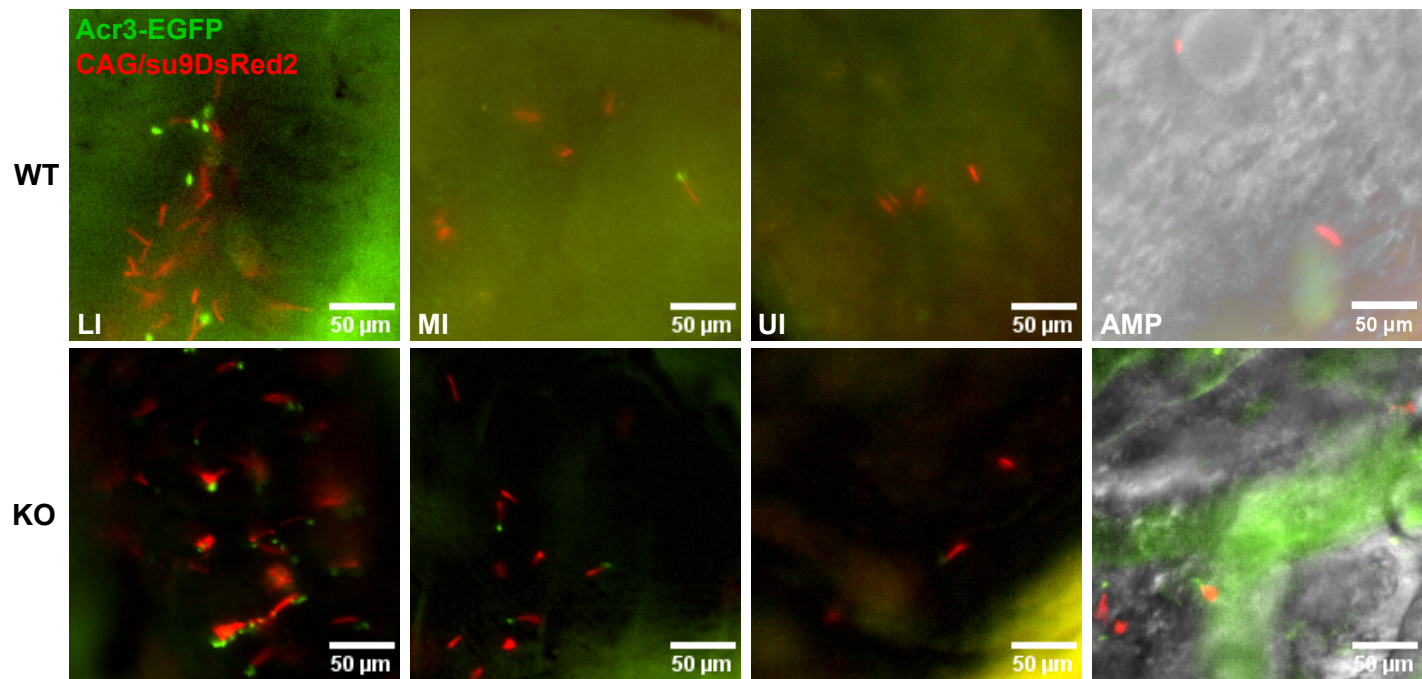

B

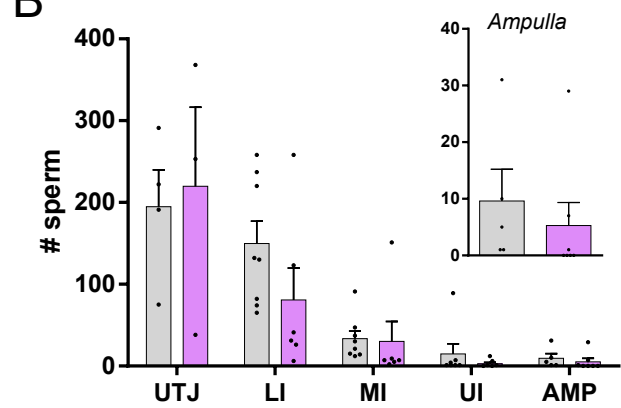

C

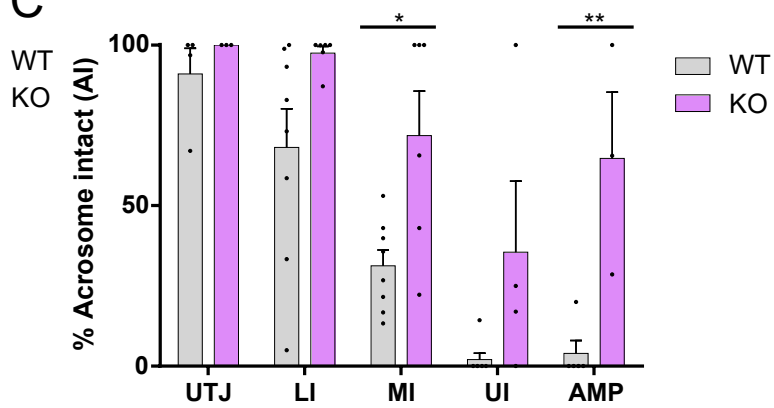

Supplementary figure 3

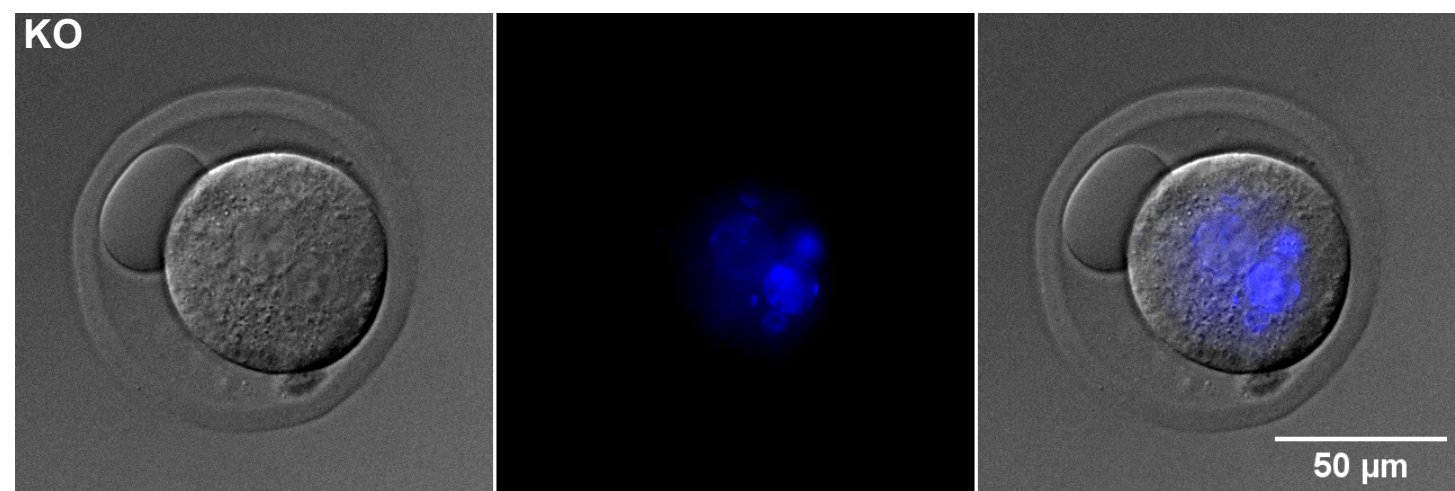

Supplement: Supplementary file 5 [file Image1.PDF]
