# Supplementary material for: Membrane potential hyperpolarization: a critical factor in acrosomal exocytosis and fertilization in sperm within the female reproductive tract
Source: Front Cell Dev Biol. 2024 May 13;12:1386980. doi: 10.3389/fcell.2024.1386980 (PMC11128623; doi:10.3389/fcell.2024.1386980)
Supplement: Supplementary file 4 [file DataSheet1.DOCX]

**Supplementary Figure legends:**

**Supplementary Figure 1: Mating behavior in EGFP/DsRed2-SLO3 male mice.** WT or SLO3 KO male mice and super-ovulated WT females were mated. Successful mating was evidenced by the presence of vaginal. Mice were mated at 5 AM and plugs were checked 40 minutes later (AM) or mated overnight (ON), the presence of sperm in the female tract was observed by epifluorescence microscopy. Values indicate the mean percentage. 5 different experiments (4 WT mice) and 6 different experiments (4 KO mice) for the AM mating. 4 different experiments (3 WT mice) and 8 different experiments (4 KO mice), for the ON mating. Chi^2^ test was performed to evaluate association between genotype and mating scheme, p= 0.094 for AM mating and p= 0.083 for ON mating.

**Supplementary Figure 2: AE in the female oviduct after ON mating.** Super-ovulated WT females were mated with EGFP/DsRed2-SLO3 WT or KO male mice. The presence of vaginal plug was used as an indicator of successful mating. Mice were mated ON and plugs were checked at 9 AM, 4 h after ovulation. **A.** Representative epifluorescence images of DsRed2 and EGFP fluorescence visible through the female tract 4 h post-ovulation. Upper panels show sperm from the WT mouse in the 4 different regions, lower *isthmus* (LI), mid *isthmus* (MI), upper *isthmus* (UI) and *ampulla* (ampulla). Lower panels show the transit of sperm from EGFP/DsRed2-SLO3 KO mice through the oviduct. **B.** Quantification of the number of sperm for WT or KO mice in the different oviduct regions, utero-tubal junction (UTJ), lower *isthmus* (LI), mid *isthmus* (MI), upper *isthmus* (UI) and *ampulla* (ampulla). Values indicate mean ± SEM of 4 different male mice for the WT and 3 different male mice for the KO. *p<0.05 stands for statistical difference between WT and KO. Multiple t-test corrected for multiple comparisons using the Holm-Sidak's method was performed. **C.** Quantification of the percentage of acrosome intact (AI) sperm of WT or KO mice in the different oviduct regions, UTJ, LI, MI, UI and *ampulla*. Values indicate mean ± SEM of 4 different male mice for the WT and 3 different male mice for the KO. *p<0.05, **p>0.01 stands for statistical difference between WT and KO. Multiple t-test corrected for multiple comparisons using the Holm-Sidak's method was performed.

**Supplementary Figure 3: Evaluation of presence of pronuclei.** Image depicting the only egg that presented 2-PN after mating super-ovulated WT females ON with EGFP/DsRed2-SLO3 KO male mice. The presence of a vaginal plug was used as an indicator of successful mating. Presence of 2-PN was assessed by epifluorescence imaging of Hoechst.
